# Computational Protein Design for COVID-19 Research and Emerging Therapeutics

**DOI:** 10.1021/acscentsci.2c01513

**Published:** 2023-03-20

**Authors:** Parismita Kalita, Timir Tripathi, Aditya K. Padhi

**Affiliations:** † Molecular and Structural Biophysics Laboratory, Department of Biochemistry, North-Eastern Hill University, Shillong 793022, India; ‡ Regional Director’s Office, Indira Gandhi National Open University, Regional Centre Kohima, Kenuozou, Kohima 797001, India; § Laboratory for Computational Biology & Biomolecular Design, School of Biochemical Engineering, Indian Institute of Technology (BHU), Varanasi 221005, Uttar Pradesh, India

## Abstract

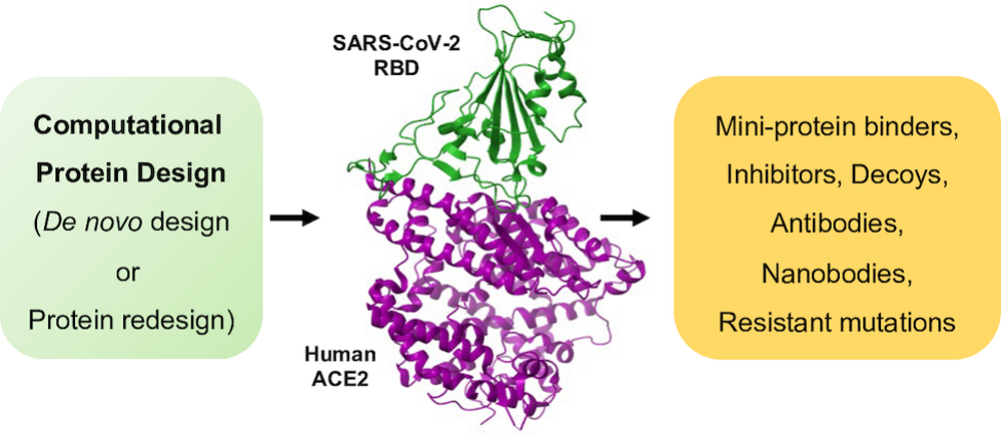

As the world struggles with the ongoing COVID-19 pandemic, unprecedented obstacles have continuously been traversed as new SARS-CoV-2 variants continually emerge. Infectious disease outbreaks are unavoidable, but the knowledge gained from the successes and failures will help create a robust health management system to deal with such pandemics. Previously, scientists required years to develop diagnostics, therapeutics, or vaccines; however, we have seen that, with the rapid deployment of high-throughput technologies and unprecedented scientific collaboration worldwide, breakthrough discoveries can be accelerated and insights broadened. Computational protein design (CPD) is a game-changing new technology that has provided alternative therapeutic strategies for pandemic management. In addition to the development of peptide-based inhibitors, miniprotein binders, decoys, biosensors, nanobodies, and monoclonal antibodies, CPD has also been used to redesign native SARS-CoV-2 proteins and human ACE2 receptors. We discuss how novel CPD strategies have been exploited to develop rationally designed and robust COVID-19 treatment strategies.

## Abstract 2

This outlook summarizes CPD approaches utilized for the development of SARS-CoV-2 therapeutics and highlights the current challenges and future directions of CPD to tackle various infectious diseases.

## 1 Introduction

Coronavirus disease (COVID-19), caused by the novel severe acute respiratory syndrome coronavirus 2 (SARS-CoV-2), is a global health concern. According to the World Health Organization (WHO) COVID-19 Dashboard (https://covid19.who.int/), this pandemic has plagued the world, resulting in over 636 million cases and 6.6 million fatalities to date. To treat SARS-CoV-2 infection, many engineered therapeutics, including vaccine candidates, antibodies, and antiviral drugs, have been designed and developed in response to this health emergency. In addition to widespread vaccination and the use of approved monoclonal antibodies and antivirals, there is still a critical need to develop new therapeutic molecules and diagnostic assays that can efficiently and affordably identify, prevent, and reduce the risk of SARS-CoV-2 infection. This is because the mutating strains pose a significant threat to the currently available vaccines and antibodies.

Because of genetic mutation and/or viral recombination, all viruses change throughout time. During this ongoing pandemic, SARS-CoV-2 has continuously evolved, accommodating various mutations that have resulted in the formation of new variants. It has disseminated across populations in diverse geographic regions. This has reduced the efficacy of the available COVID-19 therapeutics. For instance, SARS-CoV-2 Omicron subvariant BQ.1.1 has become dominant in the United States, and all FDA-approved vaccines have lost efficacy against this variant. It is estimated that the SARS-CoV-2 genome experiences 1.3 × 10^–6^ spontaneous mutations per base every infection cycle.^1,2^ In February 2020, the first SARS-CoV-2 variant with the D614G mutation in the receptor-binding domain (RBD) of spike protein was detected, and it swiftly took over as the dominant strain globally.^3^ Since then, many other variants with greater virulence and transmissibility have appeared around the world. These variants have been categorized as variants being monitored (VBM), variants of interest (VOI), variants of concern (VOC), and variants of high consequence (VOHC). The VOCs are characterized by their high transmissibility and disease severity potential and low susceptibility to different classes of therapies, antibodies, and vaccines. The currently circulating VOCs include B.1.1.529 (Omicron) and its descendent lineages BA.1, BA.1.1, BA.2, BA.3, BA.4, and BA.5.^4^ Multiple efforts are being made to develop strategies against circulating VOCs, but we must gain a deeper understanding and predict the mutational landscape of SARS-CoV-2 so that we can improve our ability to detect emerging new variants and strains of related viruses. The most transmissible Omicron variant to date (called XBB.1.5, also known as the Kraken) is currently rapidly circulating in the United States and comprises ∼50% of the cases nationwide (data from CDC Covid Data Tracker as of January 2023). According to WHO, XBB.1.5 has already been spread across 38 counties. XBB.1.5 is a combination of two Omicron BA.2 lineages with an additional S486P mutation in the spike (S)-protein compared to the XBB.1 lineage. According to preliminary studies, XBB.1.5 shows increased transmissibility and enhanced immune escape leading to reduced serum neutralizing titers in vaccinated individuals. It was also found to be resistant to neutralization by the monoclonal antibodies Evusheld and Bebtelovimab.^5−7^ Additionally, two other Omicron subvariants, CH.1.1 and CA.3.1 (with L452R mutation) are drawing attention as they continue to spread in several parts of the world, and in a recent preliminary study, they were found to be resistant to both monovalent and bivalent mRNA vaccinations.^8^ The emergence of such variants further reminds us to continuously update the current vaccine development strategies to mitigate the impact of these emerging variants on vaccine efficacy. Therefore, it should be viewed as a public health emergency to monitor the SARS-CoV-2 escape mutations, which can circumvent the effects of available vaccines, antibodies, and antivirals. We continue to live with SARS-CoV-2 as a new endemic disease. Thus, the discovery of new therapeutics that can impart prolonged protection and provide broader immunity against the present and future SARS-CoV-2 variants is the primary concern.

**Figure 1:**
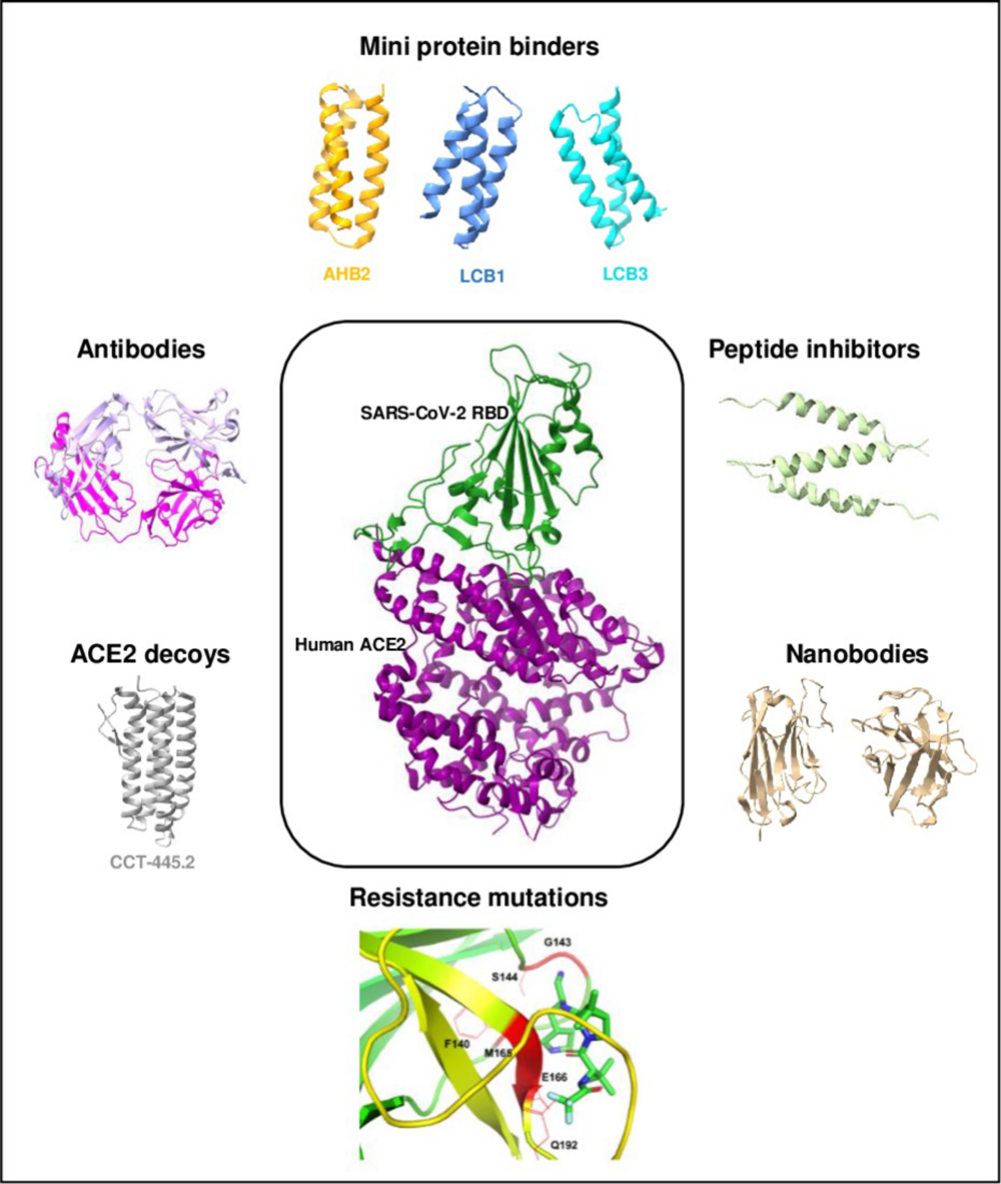
Design of miniprotein binders (AHB2, PDB ID: 7UHB; LCB1, PDB ID: 7JZU; LCB3, PDB ID: 7JZM), peptide inhibitors, antibodies, nanobodies, ACE2 decoys (CCT-445.2, PDB ID: 7KL9), and resistant mutations against SARS-CoV-2 using computational protein design approaches. Representative structures have been adapted from relevant SARS-CoV-2 studies for nanobodies, antibodies, and peptide inhibitors because PDB structures were not available for these categories.

Because computation and computational techniques have advanced exponentially in recent years, remarkable progress has been made in understanding the biology of SARS-CoV-2. Several in silico methods, computational tools, and bioinformatics resources have been used to annotate SARS-CoV-2 genomes, understand viral evolution, develop detection kits, analyze protein structures, detect potential drug targets, and develop new therapeutics. Protein design has emerged as a viable computational technique for providing alternative therapeutic strategies. Protein design technologies enable the creation of a diverse range of novel proteins with desirable properties that can be tailored for scientific, industrial, and medical applications. As a powerful method of evaluating and selecting targeted amino acid sequences on a colossal scale, computational protein design (CPD) has become a major component of biological research. Using atomic precision, one can design proteins, protein conjugates, and various types of protein molecules, which is otherwise impossible. In this outlook, we aim to understand how CPD has contributed to COVID-19 research by reducing the search/selection time for designing high-affinity protein binders and inhibitors, monitoring and predicting drug-resistance mutations, and using antibody/nanobody drugs as promising therapeutics against SARS-CoV-2 and any other emerging diseases (Figure 1).

## 2 Computational Methods Employed to Tackle COVID-19

Several experimental and computational techniques have been developed during the COVID-19 pandemic to comprehend and characterize the disease, its molecular mechanisms, and therapeutic approaches. Many of these computational or computer-aided experimental methods have resulted in the drug repurposing and identification/design of small-molecule therapeutics against SARS-CoV-2.^9^ The numerous bioinformatics tools and in silico techniques employed in SARS-CoV-2 research are covered in depth in other publications.^10−13^ The rapid identification of novel SARS-CoV-2 therapeutics has been made possible by computer-aided drug discovery (CADD) techniques, allowing the transformation of both the existing and new SARS-CoV-2-related data into a quicker experimental success.^11^ Wang et al. identified five potential molecular blockers that target the SARS-CoV-2 S-protein using in silico approaches and in vitro experiments. The blocker H69C2 had a binding affinity of 0.0947 μM and blocked viral infection in vitro with an IC_50_ of 85.75 μM.^14^ On the basis of a computational model of the HR1/2 regions and the fusion core, Ling et al. developed an antiviral peptide that specifically targets the HR1 domain of SARS-CoV-2 with a binding energy of −43.0 kcal/mol, thus blocking membrane fusion by preventing the S2 subunit from forming the prehairpin conformation.^15^ With improvements in artificial intelligence (AI) technology, many methodologies for designing small molecular inhibitors against SARS-CoV-2 have been developed. For instance, Srinivasan et al. reported an AI-based de novo design technique that used a Monte Carlo tree search algorithm (MCTS) and recurrent neural network (RNN) to develop ∼100 novel molecules against SARS-CoV-2 that outperformed existing FDA-approved molecules.^16^ A recent paper on cutting-edge technologies in the field of de novo drug design against SARS-CoV-2, employing ligand-based AI technologies, has been published elsewhere.^17^

## 3 Computational Protein Design

**Figure 2:**
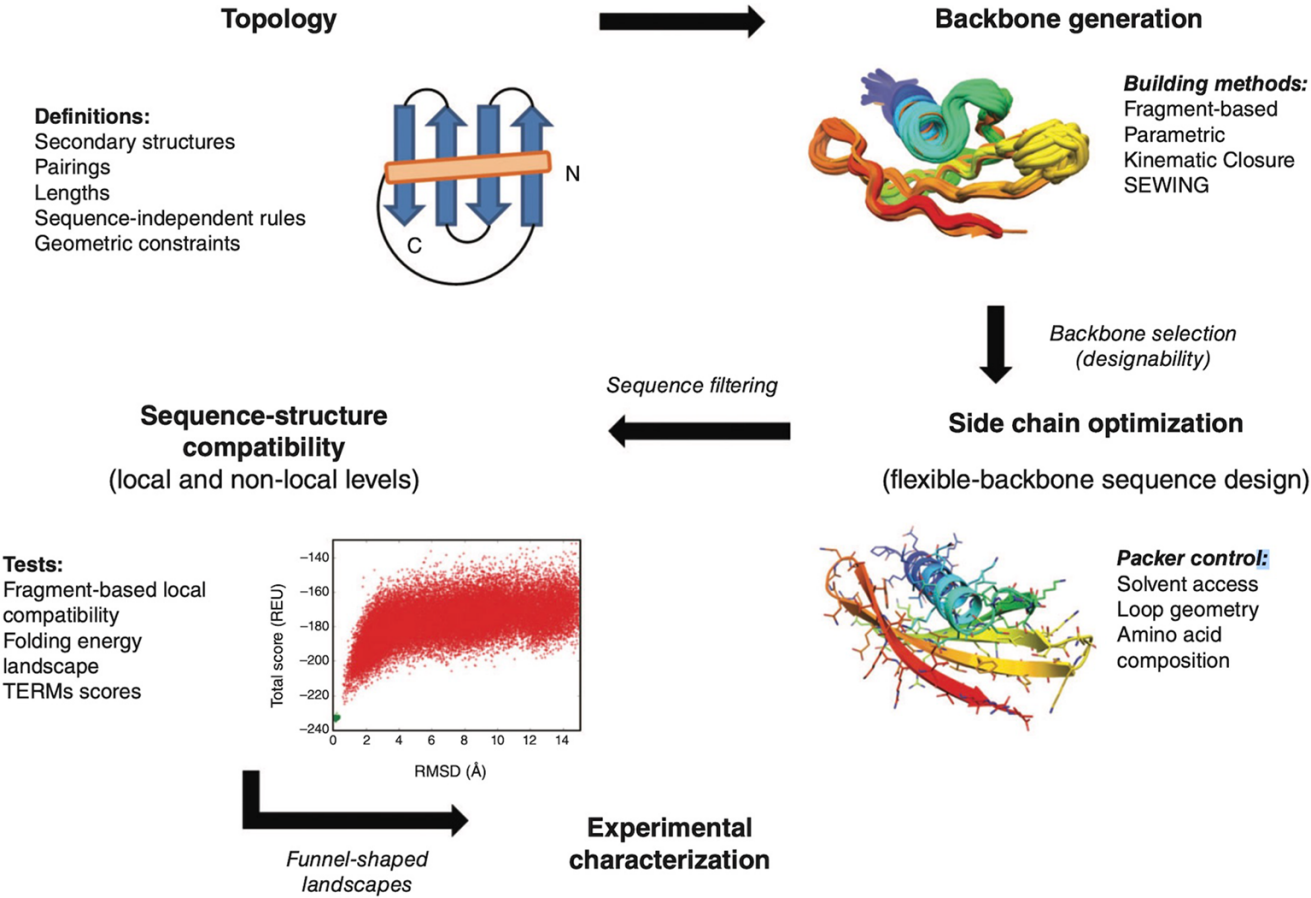
Overall workflow of de novo computational protein design. Initially, a target protein topology is defined, followed by the generation of suitable backbones to model the target topology. Compatible models are filtered, followed by an evaluation of their sequence-structure compatibility. The top-ranked backbone-sequence pairs are then selected for further experimental evaluation. Adapted with permission from ref (19) Copyright WIREs Computational Molecular Science 2018.

CPD is one of the highly regarded methodologies that have significantly contributed to strategizing alternative therapeutics for SARS-CoV-2. CPD’s superior computing capability has enabled the rapid prediction of amino acid sequences that fold into proteins with desired physicochemical properties (such as improved structural stability, binding affinity, neutralization ability, and other desired functions). Moreover, CPD combined with directed evolution has enabled the de novo design of proteins suitable for protein–ligand, protein–protein, and protein–nucleic acid interactions that do not exist in the natural proteome. The most current developments in the de novo protein design methodologies are covered elsewhere^18,19^ (Figure 2). These computationally designed proteins have been demonstrated to work in living cells through in vivo experiments. The methods and algorithms commonly employed in CPD are categorized as (i) side-chain placement, (ii) backbone conformation generation, and (iii) rigid-body placement, which typically uses classical molecular mechanics representations.^20^ CPD allows the introduction of various low-energy conformations of amino acids (called rotamers) at each position of the scaffold using the main-chain coordinates of a known protein as a scaffold. The potential mutations are sampled based on how they interact with each other and with the scaffold, ensuring fold stability with a preferred functional profile. By using optimization algorithms, amino acid sequences and rotamers are assigned energy functions to calculate the minimum interaction energy required to stabilize the fold. Despite nonconsensus, a combination of these functions favors stable protein characteristics such as hydrogen bonds, van der Waals interactions, electrostatic interactions, atomic overlap prevention, determining solvation potential, bond-dihedral potentials of a protein, etc. Additional function-specific constraints are applied to the scoring functions to enforce chemical and geometric aspects in the designed proteins to achieve the intended functionality.^21^ CPD has applications in a variety of life sciences and engineering fields, such as manipulating signaling cascades by introducing novel proteins/ligands, designing thermostable proteins with increased activity, designing self-assembling proteins to facilitate drug delivery, designing proteins with longer half-lives and novel catalytic activities, designing proteins that stimulate the immune system, etc.^20,22−28^ The subsequent sections discuss CPD’s contribution to respiratory and other viral diseases and its vast impact in alleviating SARS-CoV-2-mediated infections. An overview of various CPD approaches and methods used for different design tasks in COVID-19 and other respiratory and viral disease research is outlined in Table 1.

**Table 1:** Overview of Various CPD Approaches and Methods Used for Different Design Tasks in COVID-19 and Other Respiratory and Viral Disease Research

sl. no.	CPD approach	computational design methods	examples (target)	source
1	de novo protein design	Rosetta	BINDI, an Epstein–Barr virus (EBV) BHRF1 inhibitor	Procko et al.^29^
2	de novo folding and design	FoldFromLoops (FFL)	epitope-focused vaccine against RSV	Correia et al.^30^
3	interface and hotspot design	RosettaDesign	influenza HA stem region	Fleishman et al.^32^
4	de novo protein design	various protocols of Rosetta, such as RosettaRemodel “blueprint”, Rosetta Monte Carlo-based fragment assembly, FastDesign, Rosetta MotifGraft Mover, etc.	miniproteins against influenza hemagglutinin and botulinum neurotoxin B	Chevalier et al.^35^
5	motif transplantation and grafting	Multigraft Match, Multigraft Design	non-HIV scaffold presenting two loops from the b12 epitope against HIV gp120	Azoitei et al.^36^
6	de novo protein design and redesign	Rosetta blueprint builder, rotamer interaction field (RIF) docking	miniprotein inhibitors against SARS-CoV-2	Cao et al.^37^
7	de novo protein design and redesign	WORMS, Rosetta	trimeric miniprotein inhibitors of SARS-CoV-2	Hunt et al.^38^
8	de novo protein design	PyRosetta, Rosetta application “kcenters_clustering_of_fragments”	hACE2 decoys to neutralize SARS-CoV-2	Linsky et al.^45^
9	de novo protein design	Rosetta flex ddG, FoldX, SSIPe	hACE2 decoy against SARS-CoV-2 RBD	Havranek et al.^46^
10	redesigning	Rosetta	engineered ACE2 receptor traps against SARS-CoV-2	Glasgow et al.^49^
11	de novo protein design, interface design, grafting	EvoDesign, Rosetta Design	stable SARS-CoV-2 spike protein variants; hybrid hACE2-based peptides against SARS-CoV-2 RBD	Ong et al.,^51^ Huang et al.^52^
12	de novo protein design	CoupledMoves protocol in RosettaDesign	SARS-CoV-2-RBD peptide binders	Sitthiyotha and Chunsriviro^53^
13	protein redesign	Protein Repair One-Stop Shop (PROSS)	prefusion S-antigen against SARS-CoV-2	Williams et al.^55^
14	de novo protein design	SymPackRotamersMover in Rosetta	antibody nanocages to target SARS-CoV-2	Divine et al.^60^
15	redesigning	Rosetta	broadly neutralizing antibodies against SARS-CoV-2	Jeong et al.^61^
16	CDR grafting and redesigning	RosettaDesign	nanobodies targeting SARS-CoV-2 RBD	Yang et al.^62^
17	protein redesign	ResScan-design	identification of resistant mutation and signatures of adaptation in pathogens	Padhi and Tripathi^70^
18	protein redesign and interface-based design	Rosetta and ResScan-design	identification of favipiravir-resistant mutations in SARS-CoV-2	Padhi et al.^71^

## 4 CPD in Respiratory and Other Viral Disease Research

**Figure 3:**
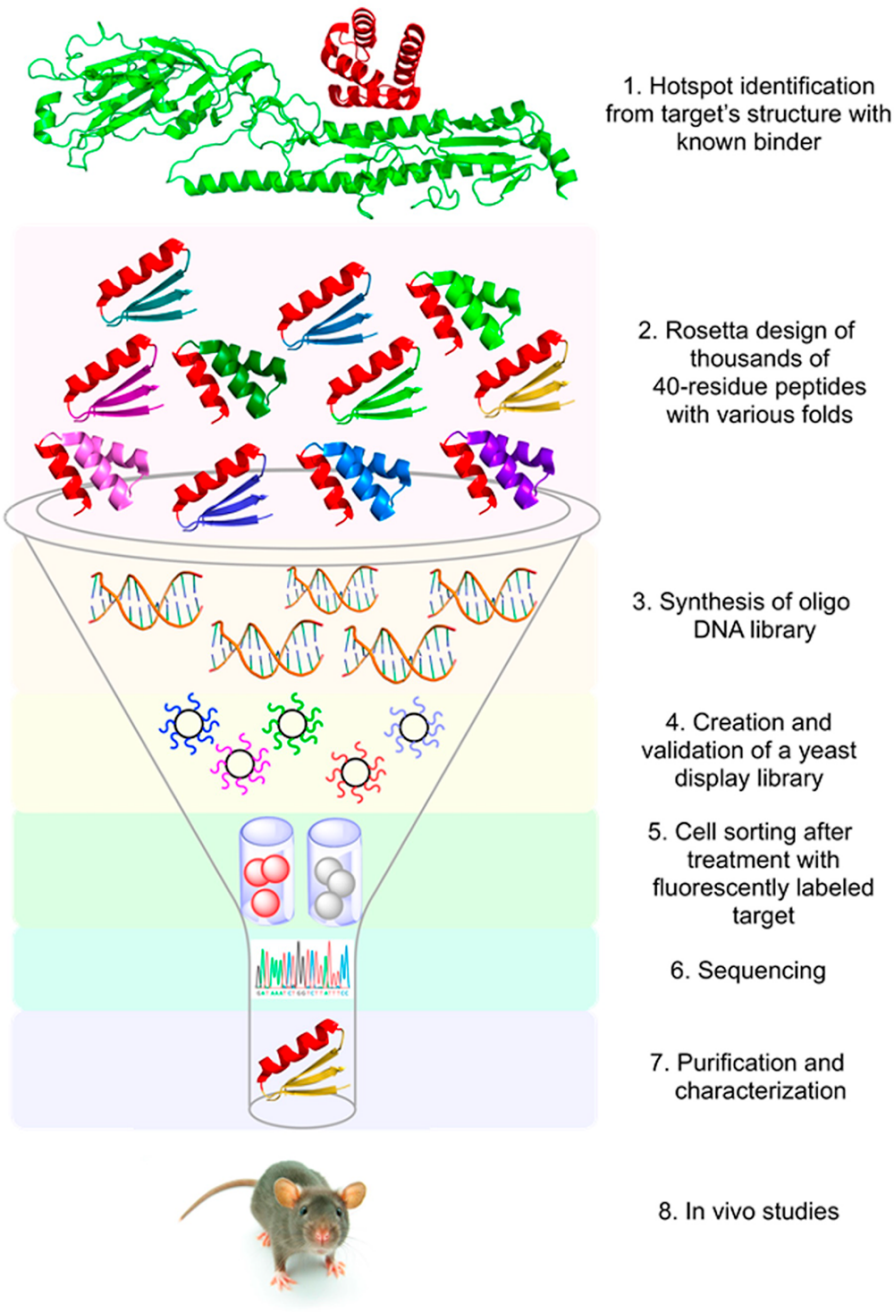
Schematic of a multistep CPD-based protein designing and high-throughput screening methodology used to produce potentially nonimmunogenic miniprotein binders. This methodology allows the identification of strong binders from a computationally designed protein library using high-throughput screening. In an ideal case scenario, steps 1–2, 3–6, and 7–8 could be performed approximately in 1–3, 3–4, and 4–6 months, respectively. It is important to note that the timeline for such integrated and multistep methodologies may vary depending on the availability of experimental/technical expertise, manpower, and access to specialized instrumentation and laboratory facilities. Adapted with permission from ref (34) Copyright Biochemistry 2017.

Numerous research groups have used CPD extensively to design novel antivirals, antibodies, or vaccine candidates against several viral infections. According to Procko et al., the effective de novo design of BINDI, a picomolar inhibitor of the Epstein–Barr virus (EBV) BHRF1 (a Bcl-2 homologue), is an excellent example of the application of the CPD approach to design therapeutic proteins. In a xenograft model of the human EBV-positive lymphoma, BINDI was able to induce apoptosis in several EBV-positive cancer lines, suppress tumor progression, and extend survivability.^29^ Using a neutralization epitope from the respiratory syncytial virus (RSV), Correia et al. designed a thermally stable protein scaffold that mimicked the viral epitope and induced neutralizing activity during immunization.^30^ An HB36.6 protein that binds the influenza hemagglutinin (HA) stem was computationally designed by Koday et al. and demonstrated to protect mice from fatal strains of the virus.^31^ Additionally, utilizing the “hotspot-design” technique, Fleishman et al. designed two high-affinity protein binders (HB36 and HB80) that specifically target the influenza HA stem region. To prevent the conformational changes in HA caused by low pH, these protein binders have nanomolar affinities for binding H1 and H5 HAs. The binding interface in the HB36 crystal structure supported their computational designs further.^32^ Chevalier et al. also presented a massively parallel de novo miniprotein creation process to design 22 660 miniproteins composed of 37–43 amino acid residues targeting influenza HA and botulinum neurotoxin B. Using Rosetta, the protein scaffolds were designed and experimentally evaluated by yeast surface display (YSD) followed by deep sequencing to detect high-affinity binders.^33,34^ The HA binder HB1.6928.2.3 was able to neutralize the influenza virus in vitro with an EC50 value similar to the FI6v3 antibody and, when administered intranasally, protected mice when exposed to a lethal dose of influenza virus^35^ (Figure 3). Using a discontinuous epitope from the HIV gp120 protein, Azoitei et al. designed a novel protein binder that binds the cross-neutralizing antibody b12 with high specificity and equivalent affinity to that of gp120.^36^

## 5 CPD in COVID-19 Research

The COVID-19 pandemic has prompted researchers to employ CPD techniques in the development of proteins, small molecules, or similar therapeutics to aid in the treatment and prevention of SARS-CoV-2 infection. Because experimental work is normally far more time-consuming and resource-intensive, the CPD approach allows for the achievement of a goal in a very short time. Although most of these designed molecules target the hACE2, S-protein of SARS-CoV-2, and the hACE2:S-protein interaction, a few are also designed to prevent viral RNA synthesis and replication, restore host innate immunity, and block other host factors or enzymes. Furthermore, numerous CPD approaches have been used successfully to predict drug-resistance mutations, possible adaption signatures of SARS-CoV-2 proteins, and their hotspot regions. The following sections discuss some significant works that used traditional and advanced CPD technologies to develop SARS-CoV-2 therapeutics.

**Figure 4:**
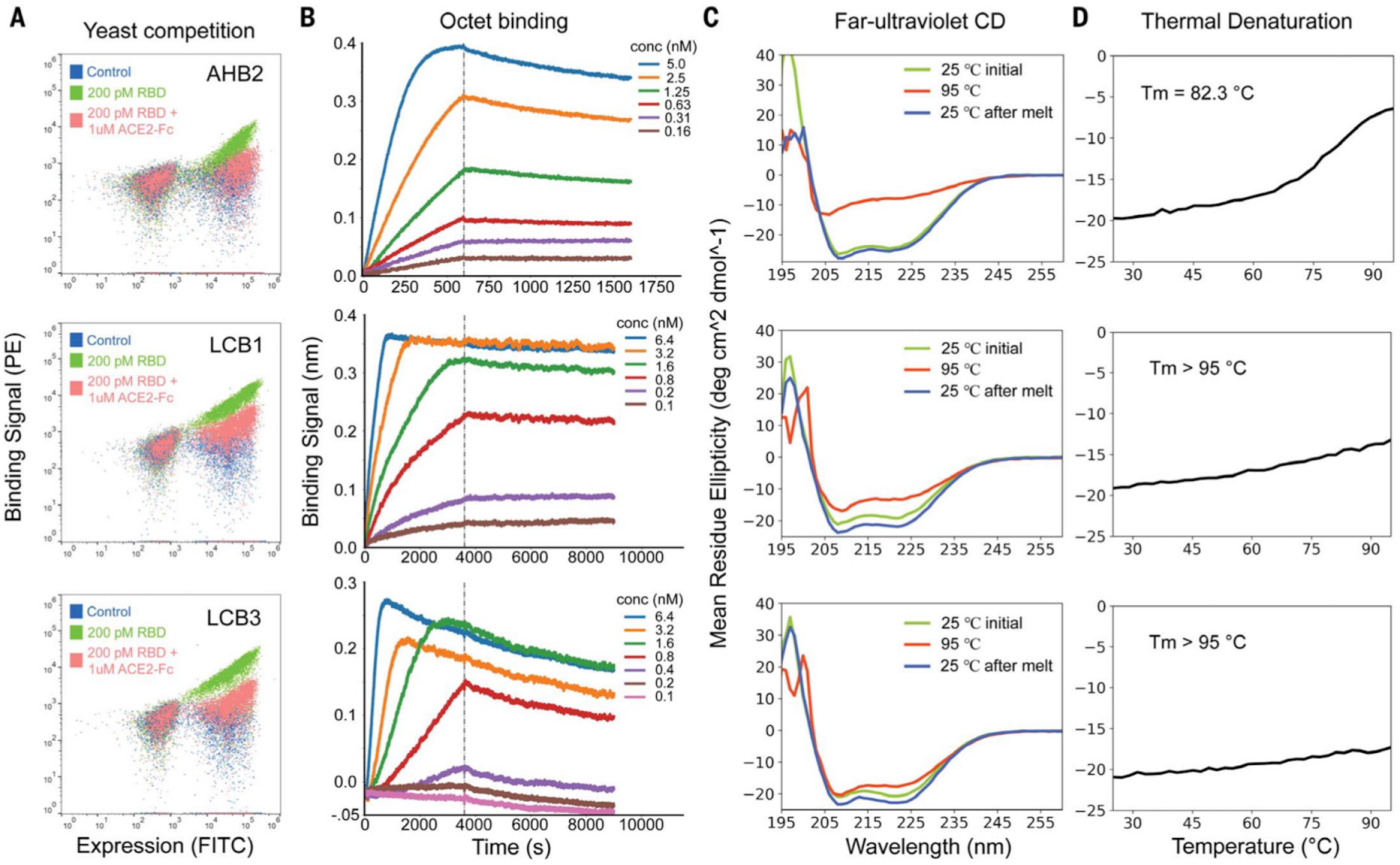
Characterization of binding and stability of the AHB2, LCB1, and LCB3 designs. (A) Flow cytometry analysis of RBD binding to yeast cells displaying the designs in the presence and absence of ACE2. (B) Binding kinetics of the designs to the RBD determined by BLI. (C) Far-UV CD spectra of the designs at different temperatures. (D) Thermal stability of the designs determined by CD. The de novo-designed proteins LCB1 and LCB3 were structurally more stable than the ACE2-scaffolded protein AHB2. Modified and adapted with permission from ref (37) Copyright Science 2020.

**Figure 5:**
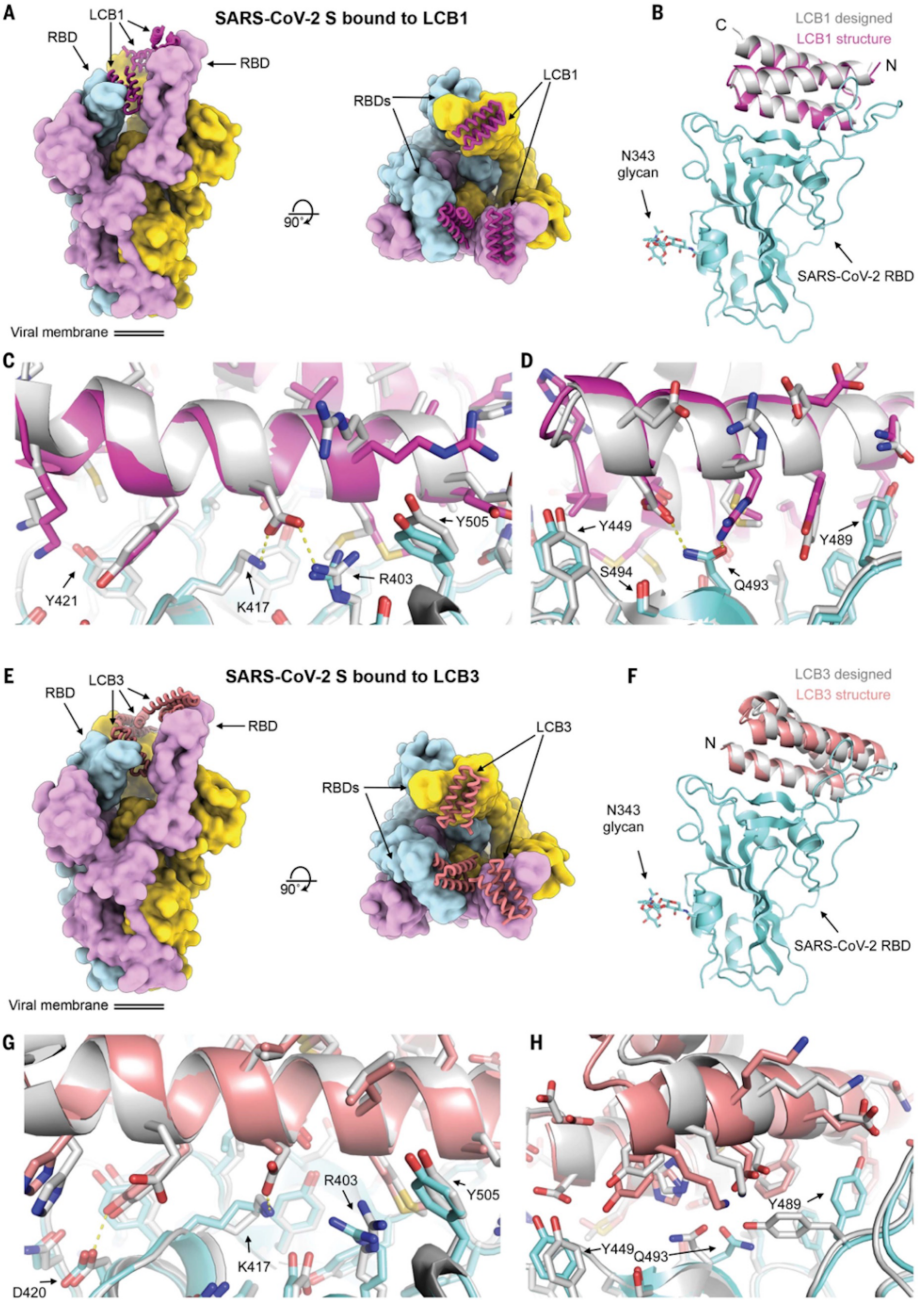
Cryo-EM analysis of the miniprotein binders. (A) LCB1 (magenta) and (E) LCB3 (pink), in complex with the RBDs of SARS-CoV-2 spike glycoprotein trimer (left, top view; right, side view). (B, F) Superimposed structures of the computational models (silver) and the cryo-EM structures of LCB1 and LCB3. (C, D) and (G, H) Zoomed-in views of the superimposed structures with the cryo-EM structures of the miniprotein binders in complex with RBD, respectively. Adapted with permission from ref (37). Copyright Science 2020.

**Figure 6:**
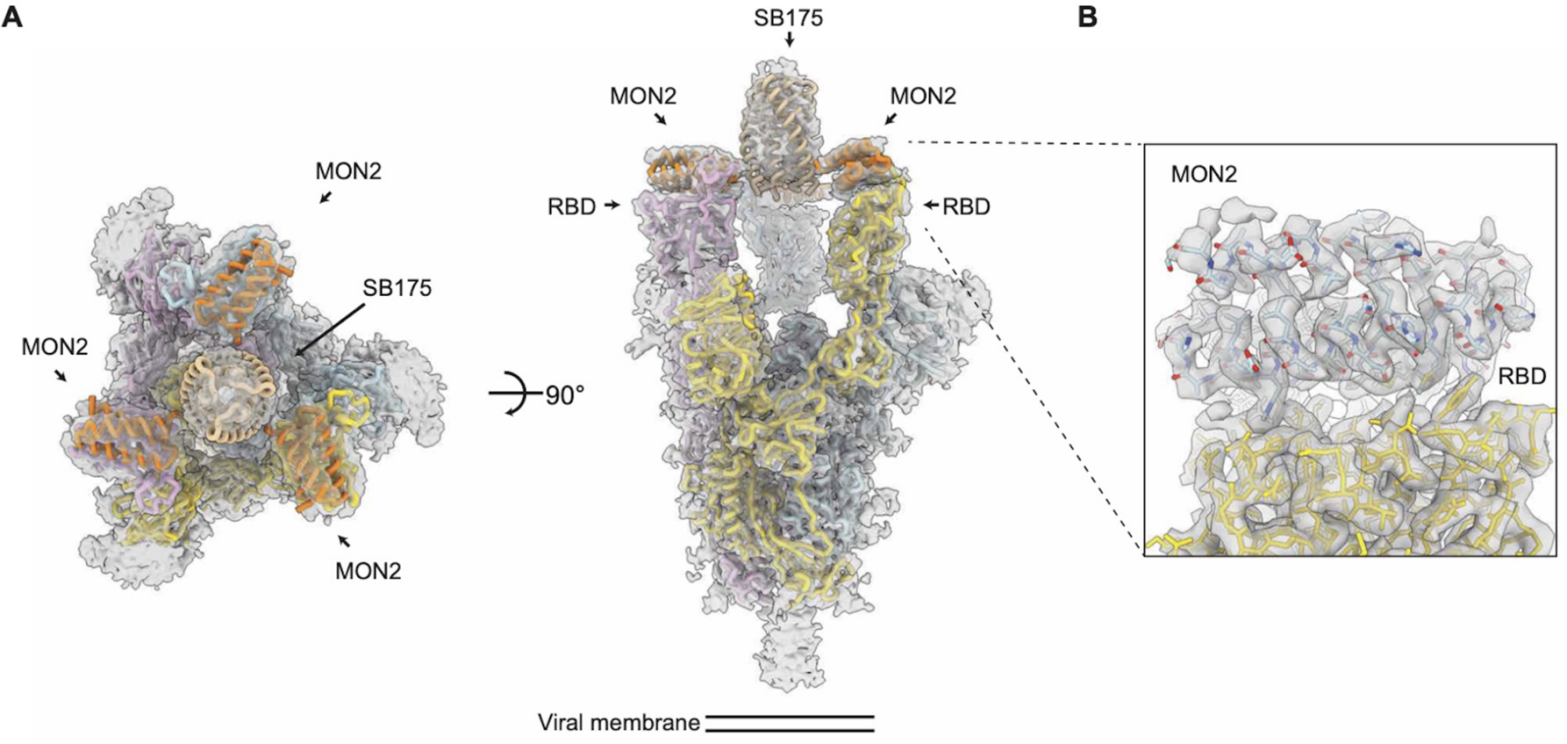
Structure of the multivalent minibinders in complex with SARS-CoV-2 glycoprotein (S6P) determined by cryo-EM. TRI2-2 is a homotrimer of MON2 generated using the SB175 homotrimization domain. (A) Top (left) and side (right) view of the TRI2-2 in complex with S6P spike glycoprotein. (B) Zoomed-in view of the TRI2-2 (blue)–RBD (yellow) complex determined by cryo-EM at a 3 Å resolution. Modified and adapted with permission from ref (38). Copyright Science Translational Medicine 2022.

Cao et al. used two different de novo design approaches to design two classes of synthetic miniprotein binders: (i) AHB2, a custom-designed three-helix bundle containing residues from the original RBD, and (ii) LCB1 and LCB3, made of entirely newly designed RBD interface residues, which showed neutralizing activity against the WA1/2020 SARS-CoV-2 virus with IC_50_ values ranging from 15 nM to 23.54 pM, respectively, in Vero E6 cells (Figures 4 and 5). These miniprotein binders are thermostable and do not require a cold chain for distribution. Besides, their small size makes them suitable for gel formulation for nasal administration and/or nebulization for direct distribution into the respiratory system. These minibinders, however, were unable to neutralize the B.1.351 (Beta) and P.1 (Gamma) VOCs.^37^ Later, Hunt et al. computationally created multivalent SARS-CoV-2-RBD specific minibinders by optimizing the previously designed LCB1, AHB2, and LCB3 minibinders. The homotrimeric minibinder TRI2-2 (a 75-residue ACE2 mimic AHB2) binds all three RBDs in a spike trimer, generating a tripod at the top of the spike protein. TRI2-2 neutralized SARS-CoV-2 VOCs and protected mice during SARS-CoV-2 challenge experiments^38^ (Figure 6). Jawad et al. also employed molecular dynamics (MD) simulations and ab initio quantum chemical calculations to develop miniprotein RBD binders with higher binding affinity than LCB1. To improve RBD binding, they added amino acid substitutions (D17R or E11V + D17R mutation) and truncated the α-helix 3 (H3) in LCB1.^39^ Furthermore, Wu et al. recently employed a similar technique to design LCB3-based miniprotein inhibitors. They designed these inhibitors by incorporating single/double/triple-point mutations in LCB3, and their best design, LCB3^H6Y-M7L-L17F^, had a binding affinity ∼45 980 times greater than LCB3.^40^ Han and Král computationally developed small peptide inhibitors and simulated them to show that they persistently and selectively bind the spike RBD. These peptide inhibitors are composed of two sequential self-supporting α-helices (α_1,2_-helixes) generated from the ACE2 protease domain (PD) and may be employed as COVID-19 treatments.^41^ Furthermore, using the protease domain of ACE2, Pei et al. designed ultrashort seven-residue peptidase inhibitors (SI5α and SI5α-b) and identified the residues from E484 to Y505 as its binding pocket on the RBD using MD simulation and binding energy analysis. These inhibitors inhibited the model coronavirus GX_P2 V, which shares 86% amino acid similarity with SARS-CoV-2-RBD.^42^

**Figure 7:**
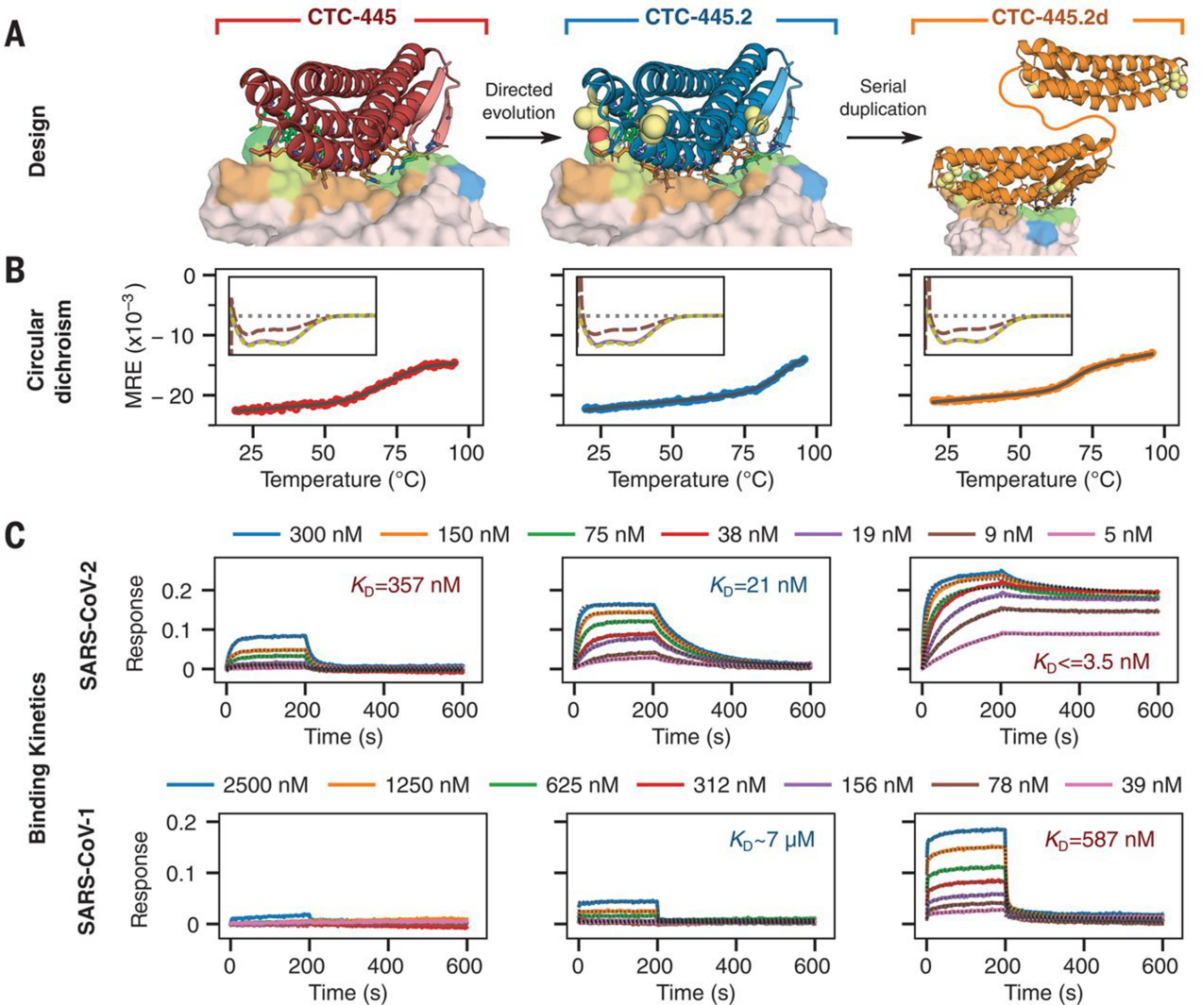
Characterization of binding and stability of the designed hACE2 decoys. (A) Design models of the de novo protein decoys CTC-445 (red), CTC-445.2 (blue), and CTC-445.2d (orange), respectively. (B) Far-UV CD spectra (at 208 nm) of the recombinantly expressed designs at different temperatures. (C) Binding kinetics of the designs to the immobilized SARS-CoV-2 RBD (top) and SARS-CoV-1 RBD (bottom) determined by BLI. Adapted with permission from ref (45) Copyright Science 2020.

Using ACE2 as the host cell receptor, a soluble ACE2 decoy protein can extensively neutralize SARS-CoV-2 variants and other sarbecoviruses.^43^ Zhang et al. reported the in vivo efficacy of an engineered ACE2 decoy, sACE2_2_.v2.4-IgG1, against SARS-CoV-2 variants in the K18-hACE2 mice model.^44^ Linsky et al. reported the de novo design of decoy proteins that mimics the hACE2 interface to bind the spike protein. The CTC-445.2 decoy (monovalent) binds all three RBDs of the SARS-CoV-2 spike protein with a nanomolar affinity (*K*_D_ ≈ 3.5 nM). In a viral challenge assay, the bivalent decoy CTC-445.2d was discovered to neutralize SARS-CoV-2 viruses in cells and protect Syrian hamsters^45^ (Figure 7). Havranek et al. used the Rosetta flex ddG method to computationally design an ACE2 decoy receptor with four mutations (ACE2-FFWF) that showed slightly higher cell surface expression and a 9-fold higher RBD binding affinity in vitro than the wild-type ACE2, as measured by flow cytometry and biolayer interferometry (BLI). An MD simulation investigation demonstrated that higher van der Waals (VDW) and hydrophobic interactions might contribute to ACE2-FFWF binding affinity.^46^ A current update on the usage of the ACE2 decoy receptor in the COVID-19 study is available elsewhere.^43,47^ Cohen-Dvashi et al. recently reported the computational design of an ACE2 immunoadhesin capable of successfully neutralizing Alpha, Beta, Gamma, and Delta VOCs.^48^ Glasgow et al. used computational design and in vitro evolution to create a high-affinity ACE2 receptor. When fused with a natural ACE2 collectrin domain and a human immunoglobulin crystallizable fragment (IgG-Fc) domain, the designed receptor had a 170-fold higher binding affinity for RBD than wild-type ACE2 and neutralized SARS-CoV-2 pseudotyped lentivirus in neutralization assays and SARS-CoV-2 virus in authentic infection studies.^49^

Zhang’s group at the University of Michigan created EvoDesign, an evolution-based de novo protein design platform (https://zhanggroup.org/EvoDesign/). This platform includes two protein design options: (a) monomer and (b) protein–protein interface design, and their design strategy comprises preprocessing, simulation, and analysis steps.^50^ Ong et al. computationally developed 22 914 spike glycoprotein variants exhibiting better immunogenicity and antigenicity. The EvoDesign algorithm was modified to create these variants with engineered MHC-II T-cell epitopes. The best candidate (Design-10705) contained nine novel T-cell epitopes, which are otherwise not present in wild-type SARS-CoV-2. However, no in vitro or in vivo experiments were reported in this work to provide experimental confirmation of the designs.^51^ Huang et al. also employed the EvoDesign algorithm to create hybrid peptide scaffolds from scratch, which were then modified to create peptides that competitively bind SARS-CoV-2 RBD, blocking virus entry into the cells.^52^ In two independent studies, Sitthiyotha et al. used CPD (using Rosetta) combined with MD simulations (employing AMBER) to create multiple 25-mer peptide binders (SBP25) of SARS-CoV-2-RBD with an increased binding affinity (higher *K*_D_). The first study used residues 21–45 of the ACE2-PD α1 helix to design SBP25 with improved *K*_D_ compared to that of the 23-mer peptide binders (SBP1) designed by Zhang et al.^44^ They developed the SBP25 by designing residues that were unknown to form favorable interactions with SARS-CoV-2-RBD to generate five peptides, namely, SPB25_F8N_, SPB25_F8R_, SPB25_L25R_, SPB25_F8N/L25R_, and SPB25_F8R/L25R_, which formed favorable interactions with SARS-CoV-2-RBD. In another study, they incorporated point mutations to these peptides to design three peptides (SPB25_Q22R_, SPB25_F8R/K11W/L25R_, and SPB25_F8R/K11F/Q22R/L25R_) with binding affinities (*K*_D_) greater than that of ACE2.^53,54^ Williams et al. recently employed Protein Repair One-Stop Shop (PROSS), an evolution-based design approach, to design a novel prefusion S-antigen, S2D14, having 20 mutations in the S2 domain of S-protein. S2D14 elicited nAbs against the SARS-CoV-2 Wuhan-Hu1 strain and four VOCs in vaccinated mice.^55^

Most monoclonal antibodies (mAbs) developed as COVID-19 prophylaxis block RBD-ACE2 binding. Mutations in the viral protein confer resistance toward these neutralizing antibodies (nAbs). For instance, the four nAbs, regdanvimab, etesevimab, casirivimab, and bamlanivimab, which were developed based on the Wuhan strain, failed to neutralize several SARS-CoV-2 VOCs (Alpha, Beta, Gamma, Delta, and DeltaPlus),^56−59^ which encouraged the development of broadly neutralizing antibodies (bnAbs). Divine et al. developed a computational methodology to design antibody nanocages by driving symmetric assembly Abs. Assembling SARS-CoV-2 antibodies into nanocages enhanced SARS-CoV-2 pseudovirus neutralization by mAbs and Fc–ACE2 fusion proteins.^60^ Jeong et al. used a computational methodology coupled with affinity maturation experiments to develop a potent bnAb that showed neutralizing potency against the current SARS-CoV-2 variants, SARS-CoV, and pangolin coronavirus.^61^

Yang et al. computationally designed 16 nanobodies by grafting complementarity-determining regions (CDRs) of SARS-CoV, MERS-CoV, and SARS-CoV-2 nAbs onto a stable nanobody scaffold. Five of 16 nanobodies were then modified to generate 7 novel nanobodies with improved stability and SARS-CoV-2 RBD-binding affinities.^62^d-Amino acid peptides are more protease-resistant, exhibit low immunogenicity, and are cost-effective. Several d-peptides have been shown to prevent HIV entry.^63,64^ Valiente et al. designed two potent ACE2 α1-binding helices that mimicked d-amino acid peptide inhibitors that bound RBD with nanomolar affinity while neutralizing the VOCs B.1.1.7 and B.1.351 in vitro and SARS-CoV-2 infection in Vero cells.^65^ Martínez et al. described an algorithm that used optimized λ-superstrings to computationally design monopeptide and multipeptide SARS-CoV-2 vaccine candidates. They combined a 22-mer peptide derived from the N-terminal domain (NTD) of the SARS-CoV-2 spike protein with a dendritic cell vector to elicit remarkable cellular and humoral immune responses; these candidate vaccines conferred a protective response in human subjects through the induction of high levels of SARS-CoV-2 neutralizing IgGs.^66,67^ Computational techniques enable the development of effective and rapid diagnostics for infectious diseases. Hajikarimlou and co-workers recently used the In Silico Protein Synthesizer (InSiPS) computational approach to aid in the design of the rapid peptide that binds SARS-CoV-2 spike protein at the RBD or the S1/S2 region, shedding more light on the field of peptide diagnostics for COVID-19 study.^68^ They designed two sets of peptides for ELISA and surface plasmon resonance (SPR) detection of SARS-CoV-2 S-protein.^69^

**Figure 8:**
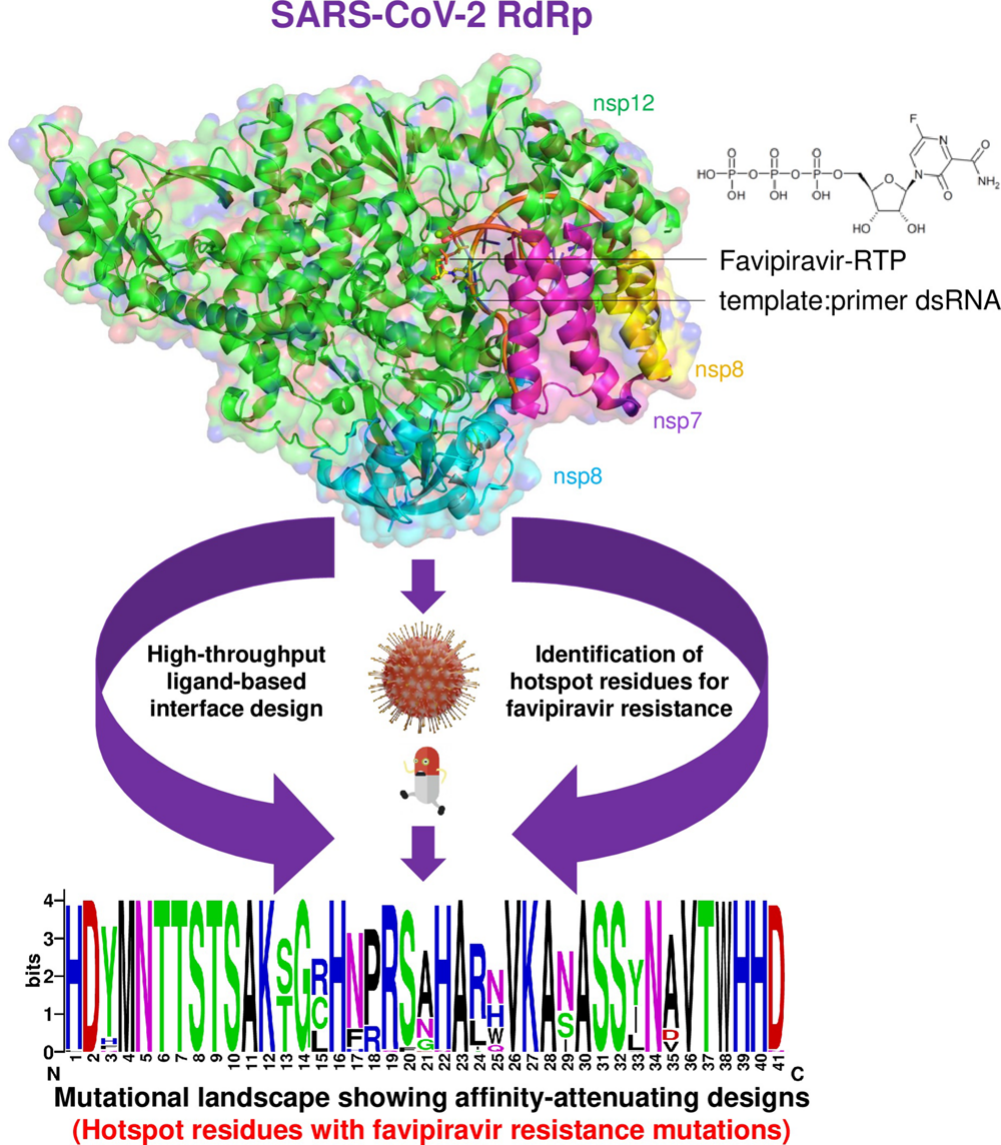
High-throughput computational protein design method used to design and predict the hotspot residues in the nsp12 subunit of SARS-CoV-2 RdRp and resistance mutations against favipiravir. The affinity-attenuating mutations in nsp12 that may contribute to favipiravir resistance are shown as the mutational landscape.

Padhi and co-workers described a unique CPD protocol, called ResScan-design, for identifying hotspot residues, resistance mutations, and adaptation signatures in a pathogenic protein against a medication, antibody, or any binding protein partner.^70^ Structure preparation using modeling tools is followed by computational protein design using the unary quadratic optimization (UQO) protein design and computation of several physicochemical properties of mutants relative to the wild-type. This methodology was utilized successfully to uncover existing circulating and susceptible resistance mutations in several SARS-CoV-2 proteins against the approved drugs for COVID-19. ResScan, for instance, was used to create and identify 13 single-point mutant designs in the SARS-CoV-2 nsp12 that are expected to develop resistance mutations against the drug favipiravir^71^ (Figure 8). They also designed resistant mutants in the SARS-CoV-2 main protease (M^Pro^) against the drugs narlaprevir,^70^ nirmatrelvir,^72^ boceprevir, and telaprevir,^73^ as well as the RNA-dependent RNA polymerase (RdRp) against the drugs favipiravir^71^ and remdesivir^74^ (Figure 8). Some of the ResScan-designed resistance mutations are already present in the circulating SARS-CoV-2 genomes, as reported in the GISAID and CoV-GLUE databases, validating the protocol’s accuracy.

## 6 Future Directions and Challenges Ahead of CPD in COVID-19 Research

CPD methods play a critical role in COVID-19 research because they hold great promise in rationalizing alternative therapeutic approaches with high success rates. The rapid improvement and adoption of data-driven AI and ML-based methods will be critical in accelerating and increasing the success rates of CPD-based therapeutics for treating and managing SARS-CoV-2. Recent advances in network and deep generative models have enabled researchers to select residues, motifs, or domains from the protein–drug or protein–protein binding sites for design experiments, allowing them to effectively use CPD for COVID-19 research. Furthermore, to improve the binding affinity and stability of engineered therapeutics, additional physicochemical features such as mutation frequency, conservation, and interactions, as well as the addition of noncanonical amino acids, may be considered. The negative design approach can be used to reduce insignificant interactions, undesirable conformations, or factors that negatively influence binding. A proper balance of positive and negative design methods, on the other hand, is important for the success of engineered SARS-CoV-2 therapeutics. Nonetheless, current CPD methods do not take into account mutations that are structurally distal from the active site or binding site, which remains a significant barrier that must be overcome. Similarly, adequate CPD methods for handling posttranslational modifications in viral proteins, such as glycosylation, which is abundant in the SARS-CoV-2 S-protein, should be developed. Furthermore, CPD software and web servers must integrate knowledge-based and energy-based methods, which may speed up the development of new therapeutics.

Intrinsically disordered proteins (IDPs) and regions (IDRs) are found in the proteome of all life forms.^75^ Around 10% of protein structures submitted in the Protein Data Bank (PDB) contain disordered regions longer than 30 amino acids,^76^ and ∼33.0% of eukaryotic proteins contain >30 residue-long disordered segments.^77^ IDPs, lacking stable tertiary structures, play numerous physiological and pathological roles, but given the dynamic and structural heterogeneity of IDPs,^78,79^ the conventional CPD approaches (specifically structure-based CPD) cannot be directly applied to such proteins/regions. Dzuricky et al. experimentally designed artificial IDPs (A-IDPs) that exhibited the phase-separation properties of biological condensates in vitro and in cells.^80^ However, the CPD-based approach remains unsubstantiated to design/engineer IDPs for therapeutic or prophylactic goals. Future attempts should be made on the IDP/IDR design using sequence-based descriptors and machine learning-based ensemble prediction with conformational transition information from order-to-disorder or vice versa routes while introducing mutations and estimating their effects by employing either de novo or physics-based methods.

The future of CPD in COVID-19 research is inextricably linked to that of directed evolution (DE). Several research groups have used the DE system to identify mutations in SARS-CoV-2 RBD that could increase its binding affinity for ACE2^81^ and produce neutralizing antibodies,^82^ nanobodies,^83^ and so on. The DE methodology, on the other hand, is labor-intensive due to the required rounds of diversification and library selection. When the sequence space is too large, the use of computational tools can simplify the process by shortening the screening time, thereby reducing the library size.^84^ The initial set of designs is created using CPD. The designs with desirable features and functions in sequence space are then subjected to successive rounds of DE experiments to determine the feasibility of the designed molecules for practical use.^20^ Protein backbone plasticity is essential for allowing conformational movements in the amino acid side chains to avoid spatial constraints. Several algorithms have been developed to add molecular flexibility and motions in the designed proteins to overcome the problems associated with the rigid and hyper-stable folds caused by the use of a fixed scaffold with limited sets of side-chain rotamers. These methodological and technological advances in CPD will be critical in developing SARS-CoV-2-engineered protein-based therapeutics.

Despite some constraints, there are tremendous opportunities in CPD that have the potential to overcome the challenges in COVID-19 research by integrating advances in theory and computation. CPD’s future potential in COVID-19 research can also be explored in other novel antiviral therapeutics with diverse practical and fundamental applications. CPD strategies have enormous potential because they are not only limited to COVID-19 research but can also be used to respond to other viruses with pandemic potential. More infectious disease outbreaks are unavoidable, and while the COVID-19 pandemic presented unprecedented challenges for researchers, it also demonstrated how new technological developments such as CPD can be explored globally for the design and development of alternative pharmaceutical products.
